# Automated airway quantification associates with mortality in idiopathic pulmonary fibrosis

**DOI:** 10.1007/s00330-023-09914-4

**Published:** 2023-07-28

**Authors:** Wing Keung Cheung, Ashkan Pakzad, Nesrin Mogulkoc, Sarah Needleman, Bojidar Rangelov, Eyjolfur Gudmundsson, An Zhao, Mariam Abbas, Davina McLaverty, Dimitrios Asimakopoulos, Robert Chapman, Recep Savas, Sam M. Janes, Yipeng Hu, Daniel C. Alexander, John R. Hurst, Joseph Jacob

**Affiliations:** 1 Satsuma Lab, Centre for Medical Image Computing, University College London, 1st Floor, 90 High Holborn, London WC1V6LJ, UK; 2 Department of Computer Science, University College London, London, UK; 3 Department of Medical Physics and Biomedical Engineering, University College London, London, UK; 4 Department of Respiratory Medicine, Ege University Hospital, Izmir, Turkey; 5 Medical School, University College London, London, UK; 6 School of Clinical Medicine, University of Cambridge, Cambridge, UK; 7 Interstitial Lung Disease Service, Department of Respiratory Medicine, University College London Hospitals NHS Foundation Trust, London, UK; 8 Department of Radiology, Ege University Hospital, Izmir, Turkey; 9 Lungs for Living Research Centre, UCL, London, UK; 10 UCL Respiratory, University College London, London, UK; 11 Respiratory Medicine, Royal Free London NHS Foundation Trust, London, UK

**Keywords:** Idiopathic pulmonary fibrosis, Lung, Mortality

## Abstract

**Objectives:**

The study examined whether quantified airway metrics associate with mortality in idiopathic pulmonary fibrosis (IPF).

**Methods:**

In an observational cohort study (*n* = 90) of IPF patients from Ege University Hospital, an airway analysis tool AirQuant calculated median airway intersegmental tapering and segmental tortuosity across the 2nd to 6th airway generations. Intersegmental tapering measures the difference in median diameter between adjacent airway segments. Tortuosity evaluates the ratio of measured segmental length against direct end-to-end segmental length. Univariable linear regression analyses examined relationships between AirQuant variables, clinical variables, and lung function tests. Univariable and multivariable Cox proportional hazards models estimated mortality risk with the latter adjusted for patient age, gender, smoking status, antifibrotic use, CT usual interstitial pneumonia (UIP) pattern, and either forced vital capacity (FVC) or diffusion capacity of carbon monoxide (DLco) if obtained within 3 months of the CT.

**Results:**

No significant collinearity existed between AirQuant variables and clinical or functional variables. On univariable Cox analyses, male gender, smoking history, no antifibrotic use, reduced DLco, reduced intersegmental tapering, and increased segmental tortuosity associated with increased risk of death. On multivariable Cox analyses (adjusted using FVC), intersegmental tapering (hazard ratio (HR) = 0.75, 95% CI = 0.66–0.85, *p* < 0.001) and segmental tortuosity (HR = 1.74, 95% CI = 1.22–2.47, *p* = 0.002) independently associated with mortality. Results were maintained with adjustment using DLco.

**Conclusions:**

AirQuant generated measures of intersegmental tapering and segmental tortuosity independently associate with mortality in IPF patients. Abnormalities in proximal airway generations, which are not typically considered to be abnormal in IPF, have prognostic value.

**Clinical relevance statement:**

Quantitative measurements of intersegmental tapering and segmental tortuosity, in proximal (second to sixth) generation airway segments, independently associate with mortality in IPF. Automated airway analysis can estimate disease severity, which in IPF is not restricted to the distal airway tree.

## Graphical abstract

**Graphical Abstract:**
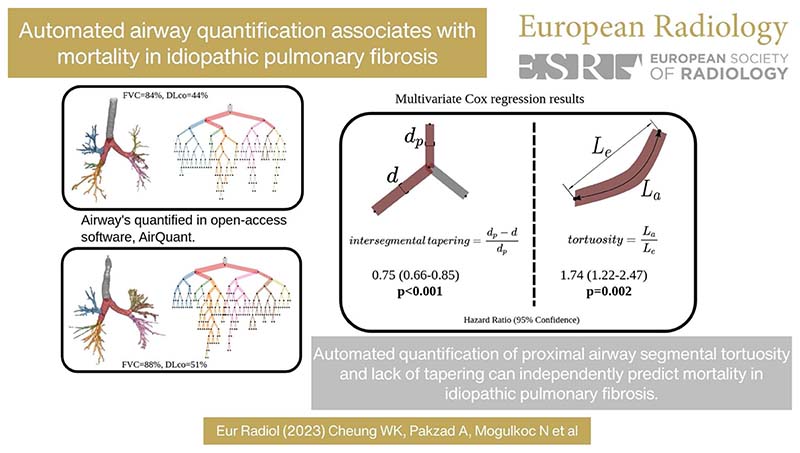

## Abbreviations

CI: Confidence interval
COPD: Chronic obstructive pulmonary disease
CT: Computerised tomography
DLco: Diffusion capacity of carbon monoxide
FVC: Forced vital capacity
HR: Hazard ratio
HU: Hounsfield unit
ILD: Interstitial lung disease
IPF: Idiopathic pulmonary fibrosis
PTK: Pulmonary toolkit
SPSS: Statistical Product and Service Solutions
STROBE: The Strengthening the Reporting of Observational Studies in Epidemiology
UIP: Usual interstitial pneumonia

## Introduction

Idiopathic pulmonary fibrosis (IPF) is a chronic progressive fibrosing lung disease diagnosed using computed tomography imaging of the lungs. A hallmark of IPF is the presence on CT of honeycomb cysts and traction bronchiectasis in a subpleural, basal lower-zone predominant distribution [1]. Traction bronchiectasis represents the pulling apart of airways walls by fibrotic contraction of the adjacent interstitial compartment of the lung. When honeycombing and traction bronchiectasis coexist in a subpleural, basal lower-zone predominant distribution [1], a patient can be ascribed a pattern of usual interstitial pneumonia (UIP). When traction bronchiectasis with the same distribution occurs in the absence of honeycomb cysts, a patient is ascribed a pattern of probable usual interstitial pneumonia (UIP) [1]. Post hoc analyses of the INPULSIS trials and other studies have shown that a UIP pattern and a probable UIP pattern have similar associations with mortality, highlighting the prognostic importance of appropriately distributed traction bronchiectasis on CT [2].

Over the past 15 years, numerous studies have examined the relationship between IPF imaging biomarkers and patient survival. Whilst these initially focussed on visual CT analysis [3, 4], recent advances in computational image analysis including machine learning and deep learning have leveraged the volumetric nature of modern IPF imaging to consider three-dimensional imaging biomarkers. Vessel-related structures comprising pulmonary arteries, veins, and associated fibrosis have been shown to associate strongly with mortality in patients across a variety of fibrosing lung diseases [5–9]. Given the prognostic importance of traction bronchiectasis when scored visually [7, 10] in patients with fibrosing lung disease, it is likely that computational measurement of airway abnormality in IPF might show promise as a prognostic biomarker.

Our study therefore aimed to examine whether automated airway metrics produced by a new airway measurement algorithm (AirQuant) associated with mortality in a population of patients with IPF. We specifically examined whether measures of the proximal airway segments of the lung (up to the sixth airway generation), which are not traditionally considered abnormal in IPF, associated with mortality.

## Materials and methods

In this observational cohort study, patients with a multidisciplinary team diagnosis of IPF and a volumetric inspiratory CT (slice thickness < 1.25 mm) were identified from Ege University Hospital, Izmir, Turkey (IPF diagnoses between 2008 and 2015). Clinical information obtained included patient age at time of CT acquisition, gender, smoking status (never versus ever), antifibrotic use (never versus ever), forced vital capacity (FVC) and diffusion capacity of carbon monoxide (DLco) obtained within 3 months of the CT, patient survival status, and follow-up time.

**Fig. 1:**
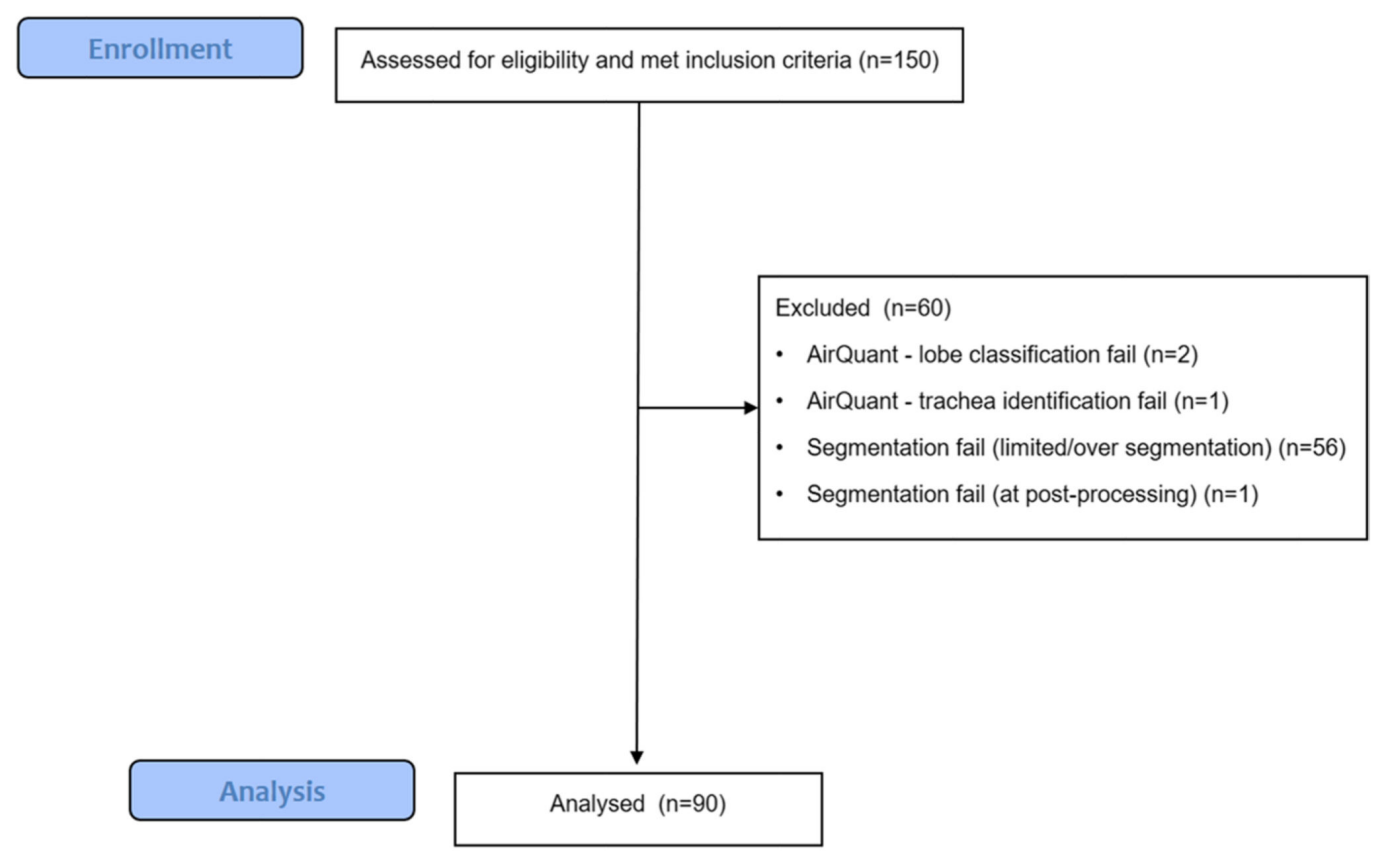
CONSORT diagram showing patient exclusions for the IPF study cohort

CT exclusion criteria included CT slice thickness > 1.25 mm, radiologic evidence of lung infection, lung cancer, and/or likely acute exacerbation on CT and CT scan quality precluding visual assessment. Patients were excluded if they had under 3 months of follow-up following the baseline CT. All cases had airway segmentation maps and lobar classifications and generational distributions visually evaluated by two observers (J.J. and A.P.) and cases deemed to have failed computationally were excluded. Computational exclusion criteria included poor airway segmentation quality, segmentation errors following use of the pulmonary toolkit, and lobe classification errors (Fig. 1). Approval for this retrospective study of clinically indicated pulmonary function and CT data was obtained from the local research ethics committees and Leeds East Research Ethics Committee: 20/YH/0120.

## Visual CT evaluation

A subspecialist radiologist (J.J.) with 15 years’ thoracic imaging experience determined lobar percentages of interstitial lung disease (ILD) (sum of ground glass density, reticulation, traction bronchiectasis volume and honeycomb cysts, averaged across six lobes [11]) and the presence of usual interstitial pneumonia (UIP) criteria [1] (definite, probable, indeterminate) on baseline CTs. The lingula lobe was considered as a sixth lobe in keeping with prior studies of fibrosing lung disease [5–9, 11, 12]. All patients with an indeterminate UIP pattern had histopathological confirmation of UIP. Traction bronchiectasis extent and severity were scored in each lobe for the first six airway generations to compare results against AirQuant. Traction bronchiectasis extent represented the number of airway segments in a lobe containing traction bronchiectasis with a maximum score of 3 in the lower lobes to avoid excess weighting in one lobe. Traction bronchiectasis extent was summed across lobes with a maximal score of 16 for the lungs (comprising a maximal score of 3 in the upper and lower lobes and 2 in the middle lobes). Traction bronchiectasis severity represented a subjective assessment of how mild, moderate, or severe the traction bronchiectasis in a lobe appeared. There was a maximal score of 3 per lobe, with a total maximal score for the lungs of 18.

## Computer-based CT evaluation

Computational airway analysis on CT imaging is a two-stage process. The first stage constitutes segmentation of the airways of the lungs. Several proprietary and open-source tools are currently available for the acquisition of an airway segmentation of the lungs. Segmentation tools can show variable performance. A reliable segmentation of the first six generations of airways is considered desirable for a segmentation tool. We developed an in-house airway segmentation tool which we used as input to our airway quantification pipeline.

### Airway segmentation

The airway segmentation algorithm combined airway segmentations obtained using the pulmonary toolkit [13] (PTK) software and an in-house deep learning segmentation which utilised a 2D dilated U-NET. The PTK software improved segmentation of the trachea and first order bronchi when compared to the deep learning model. The 2D U-NET model improved segmentation of the distal airway branches when compared to PTK. The 2D U-NET model was trained using 25 manually segmented airway trees from CTs in healthy subjects and patients with IPF. None of the CTs used to train the U-NET model was used in the current study. The airway segmentations produced by the pulmonary toolkit and 2D dilated U-NET were combined. A morphological closing procedure was then applied to re-connect airway segments that were disconnected following segmentation. The final airway tree mask was obtained by performing a largest connected components procedure.

The second stage of computational airway analysis comprises evaluation of the skeletonised airway segmentation. We have developed a computational airway tool called AirQuant which automatically identifies airway branching points and classifies the airway length between branching points into airway segments and hierarchical airway generations on a lobar basis.

### Airway quantification using AirQuant

The key aim of this study was to evaluate the performance of AirQuant. The technical details of AirQuant have been previously described [14] and are briefly summarised here. CTs < 1.25 mm were selected for analysis and a standardised window level of − 500 HU and window width of 1500 HU was used for viewing. AirQuant [14] was used to calculate airway lumen diameter and length and measurements derived from these, across all segmented airways on a CT scan. AirQuant works in a series of stages. (1) An acyclic skeleton is derived from an airway segmentation by propagating a splitting wave from the trachea [13] and applying thinning to the airways to derive an airway skeleton [15]. (2) The skeleton is converted into a graph, separating airway segments into individual components. An airway segment is defined as a single branch between splitting or endpoints. (3) Airway segments are automatically classified into their lung lobes, with the lingula interpreted as the left middle lobe. A new airway generation is produced every time an airway divides, and airway generations are counted sequentially from the trachea. Airway segments are classified according to their lobar generation. (4) Polynomial splines are used to facilitate interpolation along the branch length so that measurements can be made at intervals. This interval is dynamically set to half the size of the smallest voxel dimension of a given image. For example, if the voxel dimensions were [*x* = 0.8, *y* = 0.8, *z* = 1.0] mm, the interval would be set to measure airway segment centrelines every 0.4 mm. (5) Perpendicular airway CT slices are derived at the chosen spline interval. The size of these slices is a maximum of 40 × 40 mm, enough to frame the airway boundaries. (6) Measurements are made on these interpolated slices using the full width at half maximum edgecued segmentation limited technique [15]. An intensity profile is taken from the centre of the airway outwards at multiple angles, and the airway wall is modelled as a Gaussian curve. The inner edge of the Gaussian curve is determined to be the inner boundary of the airway lumen. An ellipse is then fitted to these boundary points. The diameter used to represent the airway at that point is computed by 2 × radius, where the radius represents the square root of the product of the minor and major axis radii.

**Fig. 2:**
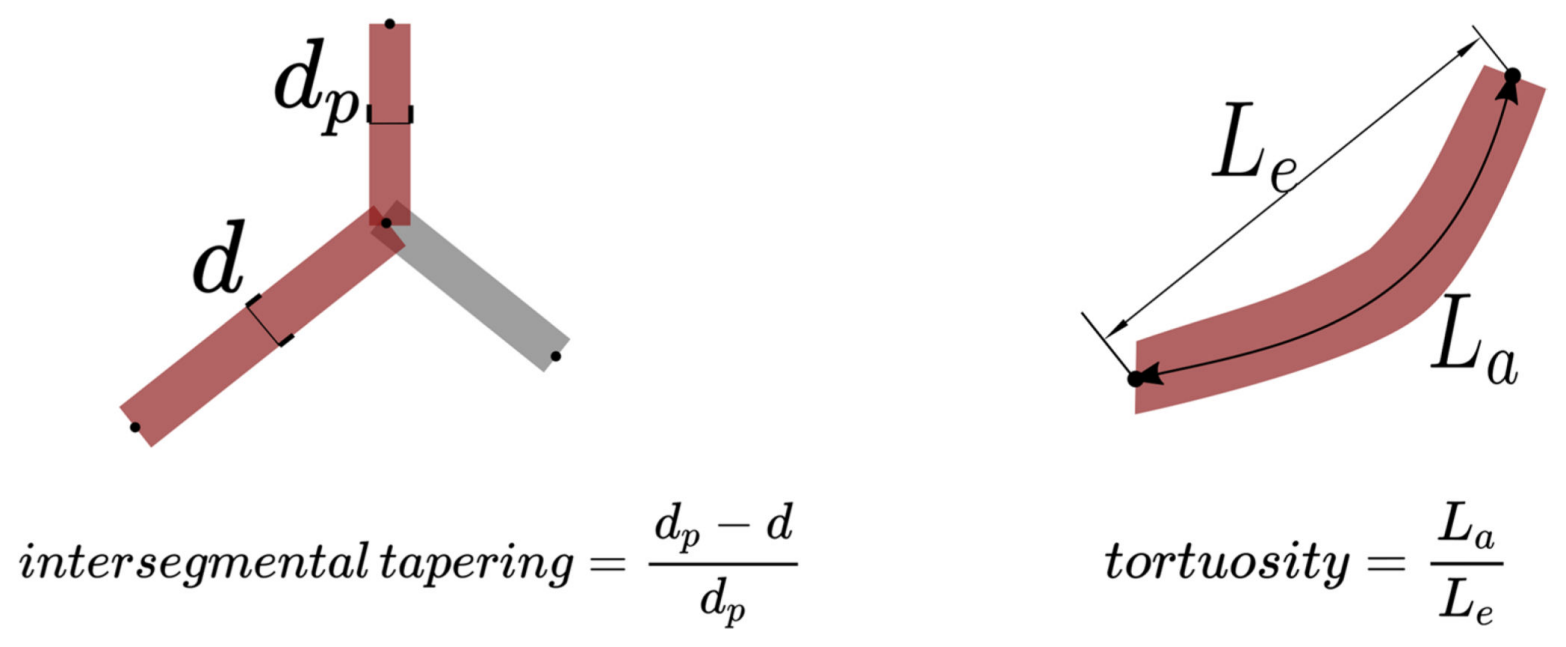
A schematic diagram illustrating the calculations used to derive intersegmental tapering and segmental tortuosity. *d_p_*, diameter of parent airway segment; *d*, diameter of child airway segment; *L_e_*, Euclid-ian airway segment length; *L_a_*, actual point to point airway segment length

**Fig. 3:**
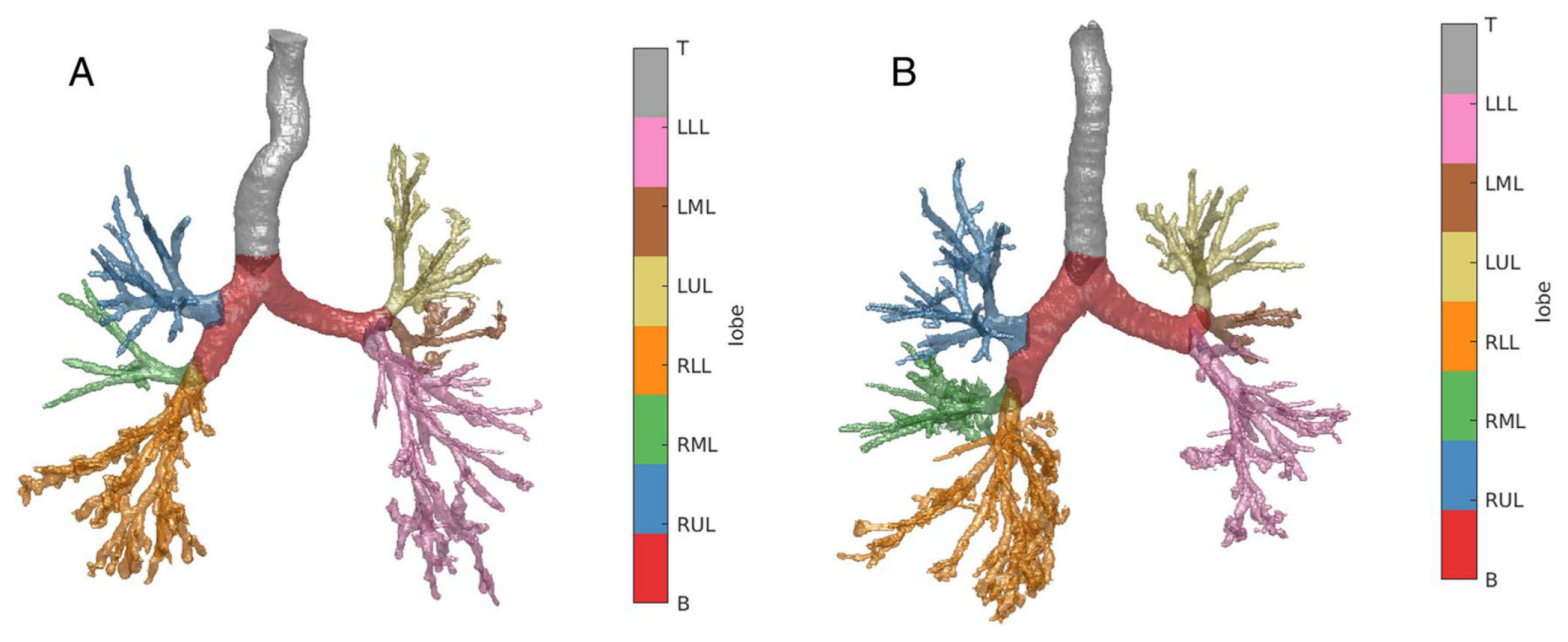
Segmentation with lobe labels of two patients diagnosed with idiopathic pulmonary fibrosis. **A** Fifty-eight-year-old, male, ex-smoker with baseline FVC = 59%, DLco = 45%, and a definite UIP pattern on CT. **B** Seventy-four-year-old, male, ex-smoker with FVC = 82%, DLco = 62%, and a definite UIP pattern on CT

**Fig. 4:**
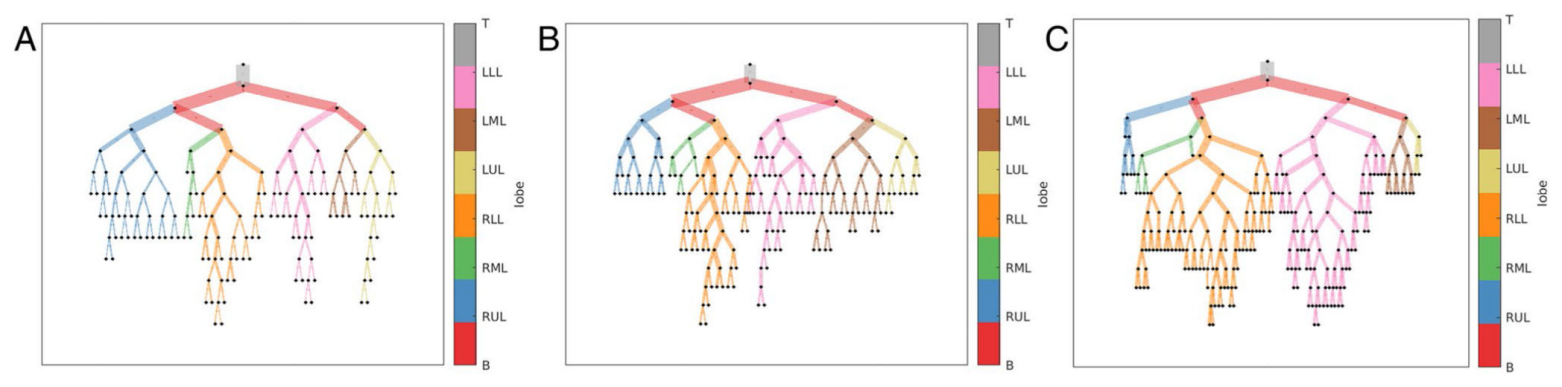
Airway graph representations of three patients **A** a 51-year-old male patient with a probable usual interstitial pneumonia pattern on CT where FVC = 84% and DLco = 44%, **B** a 79-year-old male patient with a definite usual interstitial pneumonia pattern on CT where FVC = 88% and DLco = 51%, **C** an 81-year-old male patient with a definite usual interstitial pneumonia pattern on CT where FVC = 49% and DLco = 35%. Airway divisions are represented by nodes and airway segments by edges. Edge thickness is proportional to average luminal diameter. Main and intermediate bronchi (B); edge colour represents lobe classification, RUL, right upper lobe; LUL, left upper lobe; RML, right middle lobe; LML, left middle lobe; RLL, right lower lobe; LLL, left lower lobe

Modelling the airways in this way allows systematic analysis of the airways. AirQuant variables evaluated in the study included intersegmental tapering, segmental tortuosity, and total segment count. Intersegmental tapering represents the difference in mean diameter of an airway segment and the mean diameter of its parent (proximal) airway segment, with the result divided by the mean diameter of the parent airway segment (Fig. 2). A series of diameter measurements are taken along an airway segment. The measurement intervals are set per case dependant on the voxel dimensions of a given image. A segment’s mean diameter is the mean of these diameter measurements. As airways do not taper/narrow in diameter as expected when extending into fibrotic lung, airway intersegmental tapering/narrowing would be expected to reduce. Measures of segmental tortuosity rely on calculating the Euclidean length of an airway segment. Euclidean length is determined by the arc-length between the start and end points of the airway spline (Fig. 2). The ratio of arc-length to Euclidean length (Fig. 2) denotes the degree of tortuosity of that segment, which we expect to be lower in healthy individuals. Total segment count represents the total number of airway segments identified on a CT. 3D plots of the airway tree of an IPF patient are shown in Fig. 3 and the corresponding airway graph representation is shown in Fig. 4.

The analysis considered airways in generations 2–6 in the lungs, analysed on a lobar basis.

The analysis performed here aimed to evaluate the performance of AirQuant when applied to airway segmentations trees that would be achievable by the majority of good quality airway segmentation tools currently available to researchers [16–18]. We also specifically wanted to examine whether changes in proximal airways, which are not traditionally thought to be abnormal in IPF, could provide an association with mortality.

## Statistical analysis

Data are presented as patient proportions (percentages) or means (with standard deviations) or medians (with range of values), as appropriate. Differences in categorical variables were assessed using the *χ*^2^ test. Differences in medians of continuous variables were assessed using the two-sided Mann–Whitney *U* test. Differences in means of continuous variables were assessed using the two-sided Student’s *t*-test. Univariable linear regression analyses were performed to examine relationships between AirQuant variables and clinical variables, lung function tests, and visual CT ILD extent. Univariable and multivariable Cox proportional hazards models were performed with the latter adjusted for patient age, gender, smoking status, antifibrotic use, a UIP pattern on CT, and either FVC or DLco. Separate multivariable Cox proportional hazards models adjusted for patient age, gender, smoking status, and antifibrotic use compared (a) traction bronchiectasis extent and (b) traction bronchiectasis severity with inter-segmental tapering and segmental tortuosity. Cox regression models were investigated for proportionality using plots of scaled Schoenfeld residuals. A *p*-value < 0.05 was considered significant across all analyses. Descriptive statistics, linear regression, and Cox regression analyses were performed on SPSS (version 27, IBM).

## Results

Demographic data, baseline FVC and DLco values, and visual and AirQuant CT measures for the IPF cohort (*n* = 90) are shown in Table 1. Mean patient age was 66 years with a median follow-up time of 2.7 years. Mean baseline FVC was 77.2% and mean baseline DLco was 50.6%. Patients were excluded from analysis, primarily because of segmentation failures of the airways. Excluded patients (*n* = 60) had a shorter follow-up time, more deaths, and lower baseline FVC than included patients (Table 1).

**Fig. 5:**
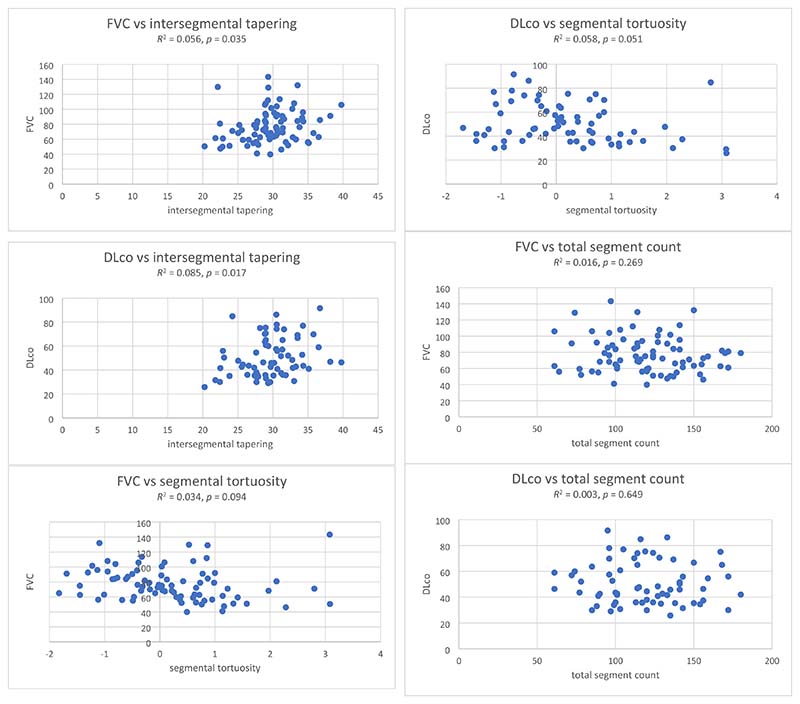
Correlations between AirQuant variables (inter-segmental tapering, segmental tortuosity, and total segment count) and baseline forced vital capacity (FVC) and diffusion capacity for carbon monoxide (DLco)

Univariable linear regression showed no significant col-linearity between AirQuant variables and patient age, gender, smoking status, antifibrotic use, visual ILD extent, visual traction bronchiectasis extent and severity, and baseline FVC and DLco (Fig. 5). On univariable Cox regression analyses, male gender, a history of smoking, no antifibrotic use, reduced DLco, increased ILD extent, increased traction bronchiectasis extent and severity, reduced intersegmental tapering, increased segmental tortuosity, and increased airway segment count associated with increased risk of death (Table 2).

On multivariable Cox regression analyses, it was notable that antifibrotic use was associated with a reduced risk of death. When considering a CT UIP pattern and disease severity adjustment using FVC, intersegmental tapering (hazard ratio (HR) = 0.75, 95% CI = 0.66–0.85, *p* < 0.001) and segmental tortuosity (HR = 1.74, 95% CI = 1.22–2.47, *p* = 0.002) independently associated with mortality (Table 3). Results were maintained with separate adjustment using DLco (Table 4). Total segment count independently associated with mortality when adjustment was made using DLco and showed a trend towards significance when adjustment was made using FVC. Intersegmental tapering and segmental tortuosity independently associated with mortality when examined in multivariable Cox regression models alongside either visual traction bronchiectasis extent (Tables 5 and 6) or visual traction bronchiectasis severity in contrast to total segment count.

## Discussion

Our pilot study has highlighted the potential for automated airway quantification, specifically intersegmental tapering and segmental tortuosity to be used as a prognostic tool in the assessment of patients with IPF. The two AirQuant metrics associated with mortality irrespective of the baseline severity of disease (as measured by FVC or DLco) and regardless of the type of UIP pattern seen on the CT.

A UIP pattern has been shown in numerous prior studies to be a strong prognostic indicator in patients with IPF [19]. Yet in our study AirQuant measures of intersegmental tapering and segmental tortuosity showed consistently stronger associations with mortality than a UIP pattern. In IPF patients, honeycombing may only constitute a small volume of the lung, and the presence of a UIP pattern therefore may not capture the extent of an individual’s disease. Disease extent may be better captured by identifying a reduction in mean tapering or an increase in tortuosity of all the airways in an entire generation in the lung.

Our observation that the proximal airways are abnormal in IPF confirm results from previous studies that have shown increased volumes [20] and reduced resistance of conducting airways in IPF [21] patients. Studies using aerosol-derived morphometry have also shown that increased airway dimensions are visible throughout the airway tree of IPF lungs [22], mirroring our finding of increased proximal airway segmental tortuosity. It is possible that proximal airway dilatation represents a potential surrogate for morphologically extensive interstitial damage on CT. However, it is notable that morphological (ILD extent) and functional measures of disease severity (FVC and DLco) only correlated weakly with AirQuant metrics of intersegmental tapering and segmental tortuosity.

One of the advantages of CT imaging over lung function measurements lies in the ability of CT to provide localised estimations of damage, whilst lung function provides averaged global measures of lung disease. Existing quantitative tools applied to the lungs have primarily quantified lung damage at a global level [23] or examined damage within lung zones [7] (upper, middle, and lower). AirQuant metrics however could provide a more granular estimate of lung damage by assessing airways at a generational level. To aid qualitative interpretation of AirQuant, airway maps were developed that demonstrate at a glance where disease is distributed in the lungs. The representation chosen was conceived with the input of pulmonologists to best mimic the airways as considered when planning bronchoscopy, and to allow patients a clear visualisation of locations of airway damage. The size of the edges in the visualisation is proportional to the diameters of the airways, which further helps delineate areas of traction bronchiectasis.

There were limitations to the current study. Though the segmentation quality of our in-house algorithm is certainly comparable to tools being used commercially, the heterogeneity of CT acquisitions and imaging constraints such as breathing artefacts made following airway courses deep into the lungs challenging for the segmentation software in certain cases. This was accentuated in patients with more severe disease who as a result had CTs excluded more frequently. AirQuant itself rarely failed (failure seen in 2% of cases) if provided with an adequate segmentation and remained robust across a variety of CT acquisition parameters, though our results will need to be confirmed in validation populations. Though this was not a study of airway segmentation performance, our results suggest that airway segmentation tools are likely to perform better in patients with early IPF and may have value in cohort enrichment of therapeutic trials [5]. We did not examine airway diameter and branch length as metrics in our analyses as these measures are confounded by patient height, age, gender, and race. Instead, we focused on parameters that indicated disease severity or which like intersegmental tapering, used an earlier generation airway in the same patient to normalise values.

In conclusion, our pilot study demonstrated that AirQuant generated measures of airway abnormality (intersegmental tapering and segmental tortuosity) significantly associate with mortality in patients with IPF. We also highlight that airway abnormalities in proximal airway generations which are not typically considered to be abnormal in IPF have prognostic value.

## Figures and Tables

**Table 1:** Patient demographics and pulmonary function indices in selected and excluded patients of IPF patients are described as mean and standard deviations, except where noted. *UIP*, usual interstitial pneumonia; *ILD,* interstitial lung disease; *FVC,* forced vital capacity; *DLco*, diffusing capacity for carbon monoxide

Variable	Selected patients (*n* = 90)	Excluded patients (*n* = 60)	*p* value
Median age (range)	66 (44–86)	66 (42–84)	0.642
Gender (male/female)	71/19	43/17	0.310
Survival (alive/dead)	50/40	18/42	0.002
Median follow-up in years (range)	2.68 (0.51–9.64)	3.43 (0.39–12.37)	0.619
Smoking (never/ever)	25/65	24/36	0.118
Antifibrotic (never/ever)	17/73	14/46	0.510
UIP (definite/probable/indeterminate)	40/50/0	32/26/2	0.098
ILD extent	42.64 + / - 12.17	50.10 + / - 13.21	0.001
FVC % predicted	77.17 + / - 22.02	66.01 + / - 19.33	0.003
DLco % predicted	50.56 + / - 16.33	46.90 + / - 21.61	0.315
Intersegmental tapering	29.48 + / - 3.84	NA	NA
Segmental tortuosity	0.18 + / - 1.00	NA	NA
Total segment count	118.19 + / - 26.93	NA	NA

**Table 2:** Univariable Cox regression models showing mortality in IPF cohorts (*n* = 90). *UIP*, usual interstitial pneumonia; *ILD,* interstitial lung disease; *FVC,* forced vital capacity; *DLco*, diffusing capacity for carbon monoxide

Variable	Hazard ratio	95% CI-lower	95% CI-upper	*p* value
Age	0.995	0.959	1.033	0.799
Gender	0.246	0.087	0.697	0.008
Smoking	2.875	1.262	6.550	0.012
Antifibrotic	0.441	0.205	0.948	0.036
ILD extent	1.049	1.021	1.078	0.001
Traction bronchiectasis extent	1.145	1.058	1.239	0.001
Traction bronchiectasis severity	1.143	1.052	1.243	0.002
UIP	0.649	0.343	1.226	0.182
FVC	0.988	0.971	1.006	0.197
DLco	0.956	0.927	0.985	0.004
Intersegmental tapering	0.816	0.745	0.895	< 0.001
Segmental tortuosity	1.543	1.119	2.128	0.008
Total segment count	1.018	1.006	1.031	0.004

**Table 3:** Multivariable Cox regression models showing mortality in IPF cohorts (*n* = 90) using intersegmental tapering, segmental tortuosity, and total segment count in airway generations 2–6. Models were adjusted for patient age, gender, smoking, antifibrotic, usual interstitial pneumonia (UIP), and forced vital capacity (FVC)

Variable	Hazard ratio	95% Cl-lower	95% CI-upper	*p* value
Age	1.003	0.955	1.054	0.901
Gender	0.244	0.051	1.154	0.075
Smoking	2.367	0.685	8.174	0.173
Antifibrotic	0.249	0.099	0.627	0.003
UIP	0.451	0.218	0.933	0.032
FVC	1.011	0.991	1.031	0.293
Intersegmental tapering	0.749	0.664	0.845	< 0.001
Age	1.024	0.975	1.075	0.348
Gender	0.151	0.033	0.692	0.015
Smoking	1.527	0.468	4.988	0.483
Antifibrotic	0.273	0.109	0.686	0.006
UIP	0.575	0.269	1.226	0.152
FVC	1.007	0.988	1.027	0.459
Segmental tortuosity	1.736	1.222	2.466	0.002
Age	1.014	0.972	1.058	0.519
Gender	0.362	0.084	1.568	0.174
Smoking	1.755	0.602	5.118	0.303
Antifibrotic	0.216	0.092	0.510	< 0.001
UIP	0.473	0.233	0.961	0.038
FVC	0.997	0.975	1.019	0.771
Total segment count	1.013	0.999	1.026	0.067

**Table 4:** Multivariable Cox regression models showing mortality in IPF cohorts (*n* = 90) using intersegmental tapering, segmental tortuosity, and total segment count in airway generations 2–6. Models were adjusted for patient age, gender, smoking, antifibrotic, usual interstitial pneumonia (UIP), and diffusing capacity for carbon monoxide (DLco)

Variable	Hazard ratio	95% Cl-lower	95% Cl-upper	*p* value
Age	1.026	0.974	1.082	0.328
Gender	0.599	0.097	3.703	0.581
Smoking	3.173	0.738	13.643	0.121
Antifibrotic	0.260	0.093	0.728	0.010
UIP	0.598	0.262	1.367	0.223
DLco	0.974	0.941	1.008	0.133
Intersegmental tapering	0.769	0.670	0.882	< 0.001
Age	1.047	0.992	1.105	0.097
Gender	0.297	0.055	1.608	0.159
Smoking	2.095	0.523	8.393	0.296
Antifibrotic	0.316	0.115	0.868	0.025
UIP	0.755	0.318	1.794	0.525
DLco	0.970	0.939	1.002	0.069
Segmental tortuosity	1.818	1.204	2.746	0.004
Age	1.012	0.971	1.054	0.582
Gender	0.484	0.125	1.877	0.294
Smoking	1.628	0.550	4.816	0.379
Antifibrotic	0.199	0.084	0.472	< 0.001
UIP	0.619	0.299	1.284	0.198
DLco	0.962	0.932	0.993	0.017
Total segment count	1.016	1.002	1.030	0.022

**Table 5:** Multivariable Cox regression models showing mortality in IPF cohorts (*n* = 90) using intersegmental tapering, segmental tortuosity, and total segment count in airway generations 2–6. Models were adjusted for patient age, gender, smoking, antifibrotic, and traction bronchiectasis extent

Variable	Hazard ratio	95% CI-lower	95% CI-upper	*p* value
Age	0.984	0.941	1.028	0.459
Gender	0.684	0.168	2.775	0.595
Smoking	3.892	1.316	11.508	0.014
Antifibrotic	0.291	0.128	0.659	0.003
Traction bronchiectasis extent	1.081	0.979	1.195	0.123
Intersegmental tapering	0.812	0.724	0.911	< 0.001
Age	0.997	0.956	1.041	0.906
Gender	0.448	0.120	1.669	0.231
Smoking	2.889	1.058	7.886	0.038
Antifibrotic	0.233	0.102	0.533	0.001
Traction bronchiectasis extent	1.117	1.018	1.226	0.019
Segmental tortuosity	1.491	1.059	2.100	0.022
Age	1.002	0.960	1.045	0.943
Gender	0.586	0.166	2.071	0.406
Smoking	2.838	1.045	7.703	0.041
Antifibrotic	0.239	0.103	0.553	0.001
Traction bronchiectasis extent	1.136	1.037	1.245	0.006
Total segment count	1.005	0.992	1.018	0.434

**Table 6:** Multivariable Cox regression models showing mortality in IPF cohorts (*n* = 90) using intersegmental tapering, segmental tortuosity, and total segment count in airway generations 2–6. Models were adjusted for patient age, gender, smoking, antifibrotic, and severity

Variable	Hazard ratio	95% CI-lower	95% CI-upper	*p* value
Age	0.985	0.942	1.029	0.498
Gender	0.637	0.157	2.590	0.528
Smoking	3.855	1.280	11.608	0.016
Antifibrotic	0.265	0.113	0.623	0.002
Traction bronchiectasis severity	1.077	0.968	1.199	0.173
Intersegmental tapering	0.815	0.725	0.915	0.001
Age	0.997	0.955	1.041	0.905
Gender	0.443	0.116	1.694	0.234
Smoking	3.071	1.080	8.732	0.035
Antifibrotic	0.200	0.084	0.477	< 0.001
Traction bronchiectasis severity	1.126	1.020	1.242	0.019
Segmental tortuosity	1.489	1.061	2.091	0.021
Age	1.002	0.960	1.045	0.940
Gender	0.633	0.172	2.333	0.492
Smoking	3.019	1.064	8.565	0.038
Antifibrotic	0.193	0.080	0.464	< 0.001
Traction bronchiectasis severity	1.148	1.045	1.262	0.004
Total segment count	1.008	0.995	1.021	0.229

## Key Points

- *AirQuant generates measures of intersegmental tapering and segmental tortuosity*.
- *Automated airway quantification associates with mortality in IPF independent of established measures of disease severity*.
- *Automated airway analysis could be used to refine patient selection for therapeutic trials in IPF*.
